# Specific Immunoassays Confirm Association of *Mycobacterium avium* Subsp. *paratuberculosis* with Type-1 but Not Type-2 Diabetes Mellitus

**DOI:** 10.1371/journal.pone.0004386

**Published:** 2009-02-10

**Authors:** Valentina Rosu, Niyaz Ahmed, Daniela Paccagnini, Gerald Gerlach, Giovanni Fadda, Seyed E. Hasnain, Stefania Zanetti, Leonardo A. Sechi

**Affiliations:** 1 Dipartimento di Scienze Biomediche, Sezione di Microbiologia, Università degli studi di Sassari, Sassari, Italy; 2 Pathogen Biology Laboratory, Department of Biotechnology, University of Hyderabad, Hyderabad, India; 3 University of Veterinary Medicine, Hanover, Germany; 4 Istituto di Microbiologia, Università Cattolica del Sacro Cuore Roma, Roma, Italy; 5 Institute of Life Sciences, University of Hyderabad Campus, Hyderabad, India; 6 Jawaharlal Nehru Centre for Advanced Scientific Research, Jakkur, Bangalore, India; BMSI-A*STAR, Singapore

## Abstract

**Background:**

*Mycobacterium avium* subspecies *paratuberculosis* (MAP) is a versatile pathogen with a broad host range. Its association with type-1 diabetes mellitus (T1DM) has been recently proposed. Rapid identification of infectious agents such as MAP in diabetic patients at the level of clinics might be helpful in deciphering the role of chronic bacterial infection in the development of autoimmune diseases such as T1DM.

**Methodology/Principal Findings:**

We describe use of an ELISA method to identify live circulating MAP through the detection of a cell envelope protein, MptD by a specific M13 phage – fMptD. We also used another ELISA format to detect immune response to MptD peptide. Both the methods were tested with blood plasma obtained from T1DM, type-2 diabetes (T2DM) patients and non-diabetic controls. Our results demonstrate MptD and fMptD ELISA assays to be accurate and sensitive to detect MAP bacilli in a large fraction (47.3%) of T1DM patients as compared to non-diabetic controls (12.6%) and those with confirmed T2DM (7.7%). Comparative analysis of ELISA assays performed here with 3 other MAP antigen preparations, namely HbHA, Gsd and whole cell MAP lysates confirmed comparable sensitivity of the MptD peptide and the fMptD based ELISA assays. Moreover, we were successful in demonstrating positive bacterial culture in two of the clinical specimen derived from T1DM patients.

**Conclusions and Significance:**

The MptD peptide/fMptD based ELISA or similar tests could be suggested as rapid and specific field level diagnostic tests for the identification of MAP in diabetic patients and for finding the explanations towards the occurrence of type-1 or type-2 diabetes in the light of an active infectious trigger.

## Introduction

*Mycobacterium avium* subspecies *paratuberculosis* (MAP) is one of the most successful pathogens of human and animals that cause chronic infection of the intestines followed by immune dysregulation resulting in painful and wasting enteritis in ruminants (Johne's disease) [1]. It is also linked to a similar type of enteritis in humans called Crohn's disease [2], [3]. While the proposed association of MAP with Crohn's disease poses a grave proposal [1], [2], [3], more alarming is its putative link to the type-1 diabetes mellitus (T1DM) [4], [5], [6]. It was long thought that the genetic susceptibilities, epitope mimicry and endemic bacterial load in the environment might support the case of an infectious trigger of T1DM in genetically susceptible individuals [4], [7], [8]. Evidence of molecular mimicry of the mycobacterial antigens with self epitopes has been revealed [9], [10], [11].

Given that the proposed infectious aetiology of T1DM is getting ground, a quick detection method that accurately and selectively identifies MAP within the diabetics is highly desirable to choose explanations between an ‘infectious’ cause or otherwise for the development of the diabetes syndrome in different individuals under different clinical settings. Polymerase chain reaction (PCR) based detection of the genetic regions of MAP such as the IS900 has been broadly successful and was recently used in diabetes cases [6] by our group; the method however needs laboratory infrastructure, and most importantly, it does not selectively detect only the live bacilli. Also, a DNA based diagnostic can not gauge immune status of the host and the development of an immune response. These issues recently lead us to the detection in diabetic patients of anti MAP antibodies [5] by using sensitive antigenic targets such as HbHA and Gsd proteins. However, these proteins are also encoded by a wider range of mycobacteria including some of the saprophytic and environmental mycobacterial organisms [12]. Further, there could be an underlying possibility of cross reactivity with the tubercle bacilli in high burden countries and in BCG vaccinated individuals. In view of these issues, search for MAP specific protein targets was intensified with some successes [13]. The identification of *mpt*D gene [14], [15] is one such effort wherein it was projected to be a MAP specific marker of virulence, expressed only on the surface of the organism and thereby a good target of the immune system. A phage specific for MptD was identified which was named as fMptD [15]. We harnessed the potential of fMptD and the MptD peptide alone for the detection of MAP in diabetic patients' blood. This study therefore, entails a novel phage based ELISA to selectively capture circulating MAP from the blood plasma with the help of MptD peptide and the fMptD. MAP cells or their products detected by MptD peptide and fMptD were further probed with anti human IgG antibodies (secondary antibodies) to detect MAP specific antibody responses in patients and controls. We propose this method to be developed for field level testing of diabetic patients for the presence of MAP; this can be employed even for high-throughput sero-prevalence studies in many different clinical case settings involving diabetes and Crohn's disease patients.

## Results

### Detection of antibodies to recombinant HbHA, Gsd and MAP antigens and whole cell lysate in T1DM and T2DM patients and controls

**Figure 1:**
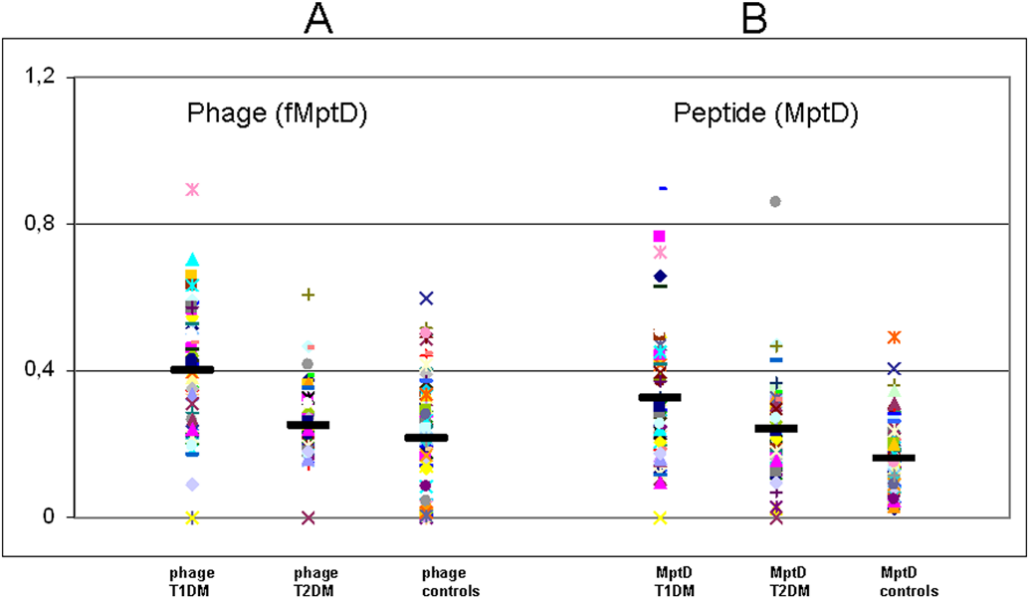
A. Evaluation of the reactivity of plasma samples representing 57 T1DM patients, 57 T2DM patients and 79 healthy controls against the fMptD (phage). B. Reactivity of plasma samples obtained from 57 T1DM patients, 57 T2DM patients and 79 healthy controls against the MptD peptide (B). Data are presented as values of OD405 observed following ELISA as described in the text. The median value for each group is indicated by a dark solid horizontal line. Data shown are from a representative experiment out of the three performed.

A significant difference among the humoral antibody responses to specific MAP antigens and whole cell lysates, as shown by the T1DM patients and the non-diabetic controls might strongly signify the involvement of MAP in T1DM (Figure 1, Table 1). The HbHA antigen gave strong ELISA titres using a cut-off value of 0.5 as previously used [6] in 58 % of the T1DM patients and only 5.1% of the controls (Chi square = 44.035, *P*<0.0001). Also, the Gsd protein revealed significant differences in ELISA titres of the diabetic (45.6% positivity) and control individuals (15.2% positivity) at the cut off value of 0.4 (Chi square = 13.74, *P*<0.0002) [5]. The cell lysate also revealed significant titres in 70% of the T1DM patients as compared to controls (7.6%) at a cut-off of 0.5 (Chi square = 54.49, *P*<0.0001) [5]. As expected, none of the results corresponding to all the three antigen preparations were comparable to the observations made for T2DM patients confirming our published data [16] (data not shown).

**Table 1:** Immune response of T1DM patients to different MAP antigens.

T1DM Patients	sex	Year of birth	Year of diagnosis	fMptD (0.4)	MptD (0.4)	HbHA (0.5)	Gsd (0.4)	MAP lysate (0.5)
01	F	1986	2000	+	+	+	−	+
02	F	1976	1996	+	+	+	+	+
03	F	1979	1985	−	+	+	−	+
04	M	1971	1982	−	−	−	+	+
05	M	1966	1989	−	−	−	−	+
06	F	1969	1997	+	−	+	−	+
07	M	1970	1996	−	−	+	−	+
08	M	1981	2005	+	+	+	+	+
09	M	1977	1996	−	−	+	−	+
10	F	1970	1998	+	+	+	−	+
11	F	1972	1985	+	+	+	−	+
12	F	1970	1970	−	−	+	−	+
13	M	1980	1989	+	+	+	+	+
14	F	1976	1979	+	+	+	+	+
15	M	1967	2005	−	−	−	−	+
16	M	1989	2003	−	−	−	−	+
17	M	1969	-	−	−	−	−	+
18	F	1967	2005	−	−	−	−	+
19	F	1976	1999	+	−	+	−	+
20	F	1981	2002	+	+	+	+	+
21	F	1971	1983	+	−	+	−	+
22	F	1967	1996	−	+	−	−	+
23	F	-	1997	+	+	+	+	+
24	F	1973	1999	−	−	−	−	+
25	M	1966	1996	+	−	+	+	+
26	F	1971	1994	−	+	−	−	−
27	M	-	1989	+	+	+	−	+
28	F	1968	2002	+	−	−	−	+
29	F	1970	1988	+	+	+	+	+
30	M	1975	1989	−	−	+	−	−
31	M	-	1983	−	−	−	−	−
32	F	1974	1984	−	−	−	−	+
33	F	1974	2002	+	+	+	+	+
34	M	1969	1994	−	−	−	−	−
35	M	1974	1995	−	−	−	−	−
36	M	1975	1976	+	−	−	−	+
37	M	1981	1994	+	−	−	−	−
38	M	1975	1988	+	+	+	+	+
39	F	1969	1974	+	−	+	+	−
40	M	1973	1989	−	−	+	+	+
41	M	-	1989	−	−	+	+	+
42	M	1966	1978	+	−	−	+	−
43	M	1913	-	−	−	+	+	+
44	M	1971	-	−	−	+	+	+
45	M	1980	2004	+	+	+	+	+
46	M	1974	1995	−	−	−	+	−
47	M	1979	2000	+	−	+	+	+
48	F	1974	1983	−	−	−	+	−
49	F	1984	1989	−	−	+	+	−
50	M	1963	1989	−	−	−	−	−
51	M	1964	1986	−	−	+	+	−
52	M	-	1992	+	−	+	+	+
53	F	1948	1979	+	−	−	+	−
54	F	1968	-	−	−	−	−	−
55	F	1965	1967	−	−	−	−	−
56	F	1968	1981	+	−	+	+	+
57	F	1960	1966	−	−	−	−	−
Total				27	17	33	26	40

### Detection of MAP by MptD-peptide and fMptD based immuno assays in T1DM and T2DM cases and controls

The MptD-peptide and the fMptD phage assay results were extremely significant and supportive of the infectious involvement of MAP in T1DM (Fig. 1). Twenty seven out of 57 T1DM plasma samples were above the cut off of 0.4 [as previously used for the other proteins [5]] when screened against fMptD, whereas, only 10 out of 79 were positive among the controls (Chi square = 18.428, *P*<0.0001). On the other hand, 17 out of 57 T1DM plasma samples were above the cut off when the MptD peptide was used in ELISA, whereas, only 2 out of 79 were positive among the healthy controls (Chi square = 17.899, *P*<0.0001). Significance of the assays was maintained when the cut-off (0.4) was calculated as 4 SD different from the mean O. D. result obtained from the negative controls (Chi square = 7.713 with 1 degree of freedom; the two-tailed *P* = 0.0055).

The results were again not verifiable in case of T2DM as shown above for the MAP antigen based ELISA results (Fig. 1, Table 2). In fact, only 4 among 57 T2DM patients were above the cut off when the phage was used in ELISA; whereas, 10 control patients were positive (Chi square = 0.279, *P* = 0.5971). Among the 57 T2DM patients, MptD based ELISA test confirmed 4 plasma read as positive; whereas, it only detected 2 positives out of the 79 controls (Chi square = 0.735, *P* = 0.3914).

Overall, the ELISA results based based on fMptD were comparable in performance and sensitivity and were superior in specificity as compared to the three antigen preparations (Figure 1, Table 1).

**Table 2:** Immune response of T2DM patients to different MAP antigens.

T2DM Patients	sex	Year of birth	Year of diagnosis	fMptD (0.4)	MptD (0.4)	HbHA (0.5)	Gsd (0.4)	MAP lysate (0.5)
01B	M	1946	2002	−	−	−	−	−
02B	F	1942	1992	−	−	−	−	−
03B	M	1942	2001	−	−	−	−	−
04B	M	1927	1989	−	−	−	−	−
05B	M	1934	2003	−	−	−	−	−
06B	M	1946	2002	−	−	−	−	−
07B	F	1958	2003	−	−	−	+	−
08B	M	1955	2005	−	−	−	−	−
09B	M	1933	2001	−	−	−	−	−
10B	F	1936	1996	+	+	−	+	−
11B	F	1968	-	−	−	−	−	−
12B	F	1932	1991	−	−	−	−	−
13B	M	1951	1998	−	−	−	−	−
14B	M	1946	1994	−	−	−	−	−
15B	F	1931	2000	−	−	+	−	+
16B	M	1952	1992	−	−	−	−	−
17B	M	1937	1993	−	−	−	−	−
18B	M	1943	2006	−	−	−	−	−
19B	M	1942	1998	−	−	−	−	−
20B	F	1939	2005	−	−	−	−	−
21B	M	1933	2004	−	−	−	+	−
22B	M	1945	2001	−	−	−	−	−
23B	F	1948	1992	−	−	−	−	−
24B	M	1940	1986	+	+	−	+	−
25B	M	1951	1996	−	−	−	−	−
26B	M	1942	-	−	−	−	−	−
27B	M	1932	2001	−	−	−	−	−
28B	F	1934	2001	−	−	−	−	−
29B	F	1932	-	−	−	−	−	−
30B	F	1944	-	−	−	−	−	−
31B	M	1939	2000	−	−	−	−	−
32B	M	1942	-	−	−	−	−	−
33B	F	1943	-	−	−	−	−	−
34B	M	1943	-	−	−	−	−	−
35B	F	1935	1992	−	−	−	−	+
36B	M	1928	-	−	−	−	−	−
37B	M	1946	1990	−	−	−	−	−
38B	M	1938	1998	−	−	−	−	+
39B	M	1934	2004	−	−	−	−	−
40B	M	1930	2003	−	−	−	−	−
41B	M	1930	2000	−	−	−	−	−
42B	F	1955	2002	−	−	−	−	−
43B	F	1959	2005	+	+	−	+	+
44B	M	1939	1993	−	−	−	−	−
45B	M	1938	2000	−	−	−	−	−
46B	M	1936	2003	−	−	−	−	−
47B	M	1948	2005	−	−	−	−	−
48B	F	1941	2000	−	−	−	−	−
49B	F	1944	1997	−	−	−	−	−
50B	M	1955	2004	−	−	−	−	−
51B	M	1952	1994	−	−	−	−	−
52B	M	1946	-	−	−	−	−	−
53B	F.	1935	-	+	−	−	−	−
54B	M	1942	1989	−	+	−	−	−
55B	F	1948	2001	−	−	−	−	−
56B	F	1946	-	−	−	−	−	−
57B	F	1953	2003	−	−	−	−	−
Total				4	4	1	5	4

The fMptD assay gave strong ELISA values (cut-off value 0.4) in 47.3 % of the T1DM patients and only 12.6% of the controls. As expected the phage assay did not detect any significant antibody values in T2DM patients (Table 2) (7% positivity) and in controls (12.6%) (Table 3).

The MptD-peptide confirmed the values obtained with the fMptD assay although with a light decrease counterbalanced by lower background levels in the controls. In particular with a cut-off value of 0.4, 29.8% of the T1DM patients tested positive and only 2.5% of the controls were found above the cut off titres (Table 3). As compared to the fMptD assay, the peptide assay did not find any significant values in T2DM patients (Table 2).

**Table 3:** Immune response of control subjects to different MAP antigens.

Controls	sex	Year of birth	fMptD (0.4)	MptD (0.4)	HbHA (0.5)	Gsd (0.4)	MAP lysate (0.5)
01C	F	1974	−	−	−	−	−
02C	F	1964	−	−	−	−	−
03C	M	1982	−	−	−	−	−
04C	F	1957	−	−	−	−	−
05C	M	1977	−	−	−	−	−
06C	F	1978	−	−	−	−	−
07C	F	1969	−	−	−	−	−
08C	M	1950	−	−	−	−	−
09C	F	1947	−	−	−	−	+
10C	M	1962	−	−	−	−	−
11C	M	1962	−	−	−	−	−
12C	M	1958	−	−	−	−	−
13C	M	1970	−	−	−	−	−
14C	M	1974	−	−	−	−	−
15C	M	1946	−	−	−	−	−
16C	M	1946	−	−	−	−	−
17C	M	1946	−	−	−	−	−
18C	F	1947	−	−	−	−	−
19C	M	1964	−	−	−	−	−
20C	F	1973	−	−	−	−	−
21C	F	1982	−	−	−	−	−
22C	M	1950	−	−	−	−	−
23C	M	1962	−	−	−	−	−
24C	F	1979	−	−	−	−	−
25C	M	1962	−	−	−	−	−
26C	M	1962	+	−	−	−	−
27C	M	1970	−	−	−	−	−
28C	M	1959	−	−	−	−	−
29C	F	1950	−	−	−	−	−
30C	M	1977	−	−	−	−	−
31C	M	1970	+	+	+	+	+
32C	M	1968	−	−	−	−	−
33C	M	1979	−	−	−	−	−
34C	F	1972	+	−	−	−	−
35C	F	1986	−	−	−	−	−
36C	M	1986	−	−	−	−	−
37C	M	1962	−	−	−	−	−
38C	M	1968	−	−	−	+	−
39C	F	1961	−	−	−	+	−
40C	M	1988	−	−	−	−	−
41C	M	1971	−	−	−	−	−
42C	F	1956	−	−	−	−	−
43C	M	1983	+	−	−	+	+
44C	M	1965	−	−	−	−	−
45C	M	1946	+	−	−	+	−
46C	F	1974	−	−	−	+	+
47C	M	1978	−	−	−	−	−
48C	M	1954	−	−	−	−	−
49C	M	1983	+	−	−	−	−
50C	M	1984	+	−	−	−	+
51C	F	1979	−	−	−	−	−
52C	M	1972	−	−	−	+	−
53C	M	1986	+	−	−	+	+
54C	M	1979	−	−	−	+	−
55C	M	1978	−	−	−	−	−
56C	M	1984	−	−	−	−	−
57C	F	1965	−	−	−	−	−
58C	F	1969	−	−	−	−	−
59C	M	1974	−	−	−	−	−
60C	M	1979	−	−	−	−	−
61C	M	1963	+	−	−	−	−
62C	M	1966	−	−	−	−	−
63C	F	1978	−	−	−	−	−
64C	F	1979	−	−	−	−	−
65C	M	1958	−	−	−	−	−
66C	M	1981	−	−	+	+	−
67C	M	1978	−	−	+	−	−
68C	M	1965	−	−	−	−	−
69C	M	1975	+	−	+	+	−
70C	M	1958	−	−	−	+	−
71C	M	1950	−	−	−	−	−
72C	F	1968	−	−	−	−	−
73C	M	1983	−	−	−	−	−
74C	M	1976	−	−	−	−	−
75C	F	1974	−	−	−	−	−
76C	M	1976	−	−	−	−	−
77C	M	1980	−	+	−	−	−
78C	M	1983	−	−	−	−	−
79C	M	1964	−	−	−	−	−
total			10	2	4	12	6

We do not believe that the background associated with other mycobacterial loads might have affected (or augmented) our results. This is because the tuberculosis status of all our subjects and controls was definitely negative. Also, since the fMptD and the MptD assays are based on blood plasma, we seemingly assayed presence of live bacilli (and their products) in the blood of T1DM patients, which was certainly the MAP, because from two out of ten T1DM inoculated samples presence of MAP cells was confirmed by growth as well as by amplification of IS900 as a MAP specific target [6], [18]. Another important reason to argue in favour of the specificity of the test is that it is specific to a mycobacterial envelope protein MptD, which is uniquely present only in MAP [14], [15]. Moreover, when the phage alone (not codifying for the MptD protein) was used as a control, the results revealed no statistical difference between T1DM and control plasma as well as T2DM and control plasma (data not shown).

### Isolation of MAP from T1DM plasma samples

Ziehl-Neelsen staining performed on the T1DM, T2DM and control samples was negative for the presence of any acid fast cells. However, after 14 weeks MGIT instrument detected growth in 2 (n. 23 and 27) of the 10 inoculated T1DM samples but none in the other samples. An aliquot of the MGIT culture was then inoculated onto 7H10 solid medium plus mycobactin J and after 6 weeks small colonies were observed. None of the other samples were tested positive for bacterial growth after 6 months and then they were discarded. Samples 23 and 27 were also both positive for all the serological tests used except for Gsd which was not recognized by the serum of patient no. 27.

The 2 isolates were not able to grow in 7H10 without the addition of Mycobactin J and they tested positive for IS900 (MAP species specific) PCR previously used [6], [18]. Sequence of the amplicon confirmed IS900 identity (data not shown).

## Discussion

The Mediterranean island of Sardinia is highly endemic for the presence of MAP infection in sheep [3], [17] concurrently with a very high number of MAP positive Crohn's disease patients [3], [17], [18]. Infected domestic animals such as sheep and cattle excrete huge numbers of MAP into the environment where their survival and persistence particularly within the protists is well established [1], [19]. Environmental cycling involves spread of MAP through wildlife such as rabbits and re-infection of livestock also through deposition of extracted slurry from water treatment plants back onto the farmland [1], [19]. Further, there is a potential for the dispersal of these pathogens via aerosols and for the cycling of human strains of MAP within human populations [19]. Passage of these robust, versatile organisms within such cycles extends the opportunity for their evolution and augmentation of pathogenicity. Humans are exposed to MAP through the environment and after the community milk supplies get contaminated [1], [2]. Influenced by the phenotype and genetic lineage of the organism the outcome of such exposure may be the acquisition of some natural resistance. In other cases it may lead to persistent colonization which, in the presence of particular virulence determinants coupled with host susceptibility, may eventually lead to the development of chronic inflammation of the intestines with features typical of paucimicrobial Johne's disease in animals [2], [3]. The reliable scientific evidence for the involvement of strains of MAP in the causation of Crohn's disease has become very strong in recent times [2], [3], [19]. Common conditions such as irritable bowel syndrome, as well as others, are on the list of additional associated conditions [20]. The MAP problem presents as a covert, slowly evolving epidemic whose progression with all its threats involving humans and farm animals, will continue until the true nature of the threat is at last unraveled. The well-argued case of T1DM [5] is just another facet of this threat which is looming large.

T1DM develops as a consequence of autoimmune responses that lead to the destruction of insulin producing beta cells of the pancreas [7]. Also, there has been a long speculation over the involvement of an infectious trigger underlying such autoimmune responses [4], [9], [11], [21], [22], [23], [24]. Recent evidences originating from the previous studies from our group associate MAP infection to the T1DM through PCR and ELISA based qualitative detection [5], [6].

There is however, the need to prove presence of live, circulating bacteria in diabetic patients that might potentiate the idea of a persistent infection existing in such patients as a first step to confirm involvement of MAP in diabetes. It was previously shown in case of *M. tuberculosis* that they often escape to the peripheral blood in case of invasive pulmonary tuberculosis [25]. Also, MAP were successfully cultured from the blood of Crohn's disease patients [2]. Circulation of MAP in diabetics was speculated [11] based on PCR assay, but, PCR could also detect degraded DNA as the products of lysed bacilli [6]. The immunogenic proteins of diagnostic value, namely the HbHA and Gsd that we successfully used to demonstrate anti-MAP humoral responses in the past suffer up to some extent because they are not MAP specific in a strict sense [5] and again they may not be indicative of an active infection. We therefore, sought to develop a method that was competent to specifically trap live MAP bacilli from blood since culture of MAP is quite tedious and difficult and often bacillary load in the samples in the absence of an invasive disease like Crohn's may not be sufficient to generate optimum growth in culture media [2], [3], [26]. Our fair choice therefore, was limited to a phage based detection and we narrowed down our option to a MAP specific protein MptD, for which a specific phage was fortunately available [15]. The phage fMptD binds on the surface of MAP and its target, the MptD is already an established virulence factor that expressed during active infection of the natural host [14], [27]. Recently it was validated as a diagnostic target for the detection of MAP in a large number of bulk milk samples obtained from MAP infected herds [15].

Two different types of ELISA were performed – first, by fixing the phage to the plates and testing against the plasma of patients and controls. Here, if MAP was present in the plasma it was binding to the phage through MptD. Given the fact that in the plasma of patients (where MAP is present), antibodies against MAP shall be present and these antibodies are likely to bind to the phage-trapped MAP cells. In this case, secondary anti human IgG conjugated with alkaline phosphatase shall bind to the anti MAP previously bound. So the signal was amplified after adding the enzyme substrate. In the second format we coated the plates first with the plasma of patients and controls and then added fMptD. The fMptD was probed by an enzyme conjugated anti M13 antibody. In this case we obtained the same results but with a lower sensitivity; this may be because we are revealing only one target (MptD), whereas, in the first case although the binding was MptD specific the results were indicative of an amplified signal from different anti MAP antibodies present in the plasma of positive patients. Both the formats (fMptD and MptD ELISA) thus supported our hypothesis that MAP is circulating in the blood of T1DM patients.

The isolation of two MAP strains out of ten inoculated fresh blood samples is extremely important. It supports the results obtained by the MptD ELISA confirming our hypothesis that live MAP are circulating within the blood stream of T1DM patients. Furthermore, the T1DM patients from whom the MAP strains were isolated (23 and 27), were also both positive for all the serological tests used except for Gsd (antibodies for which were not abundant in the plasma of patient no. 27); this is a further indication of the accuracy of the tests that we employed.

The fact that the phage performed better in the T1DM patients is counterbalanced by the relatively high level of positivity found in the controls. Perhaps a relative lack of specificity for the phage assay (instead of an increased sensitivity of the phage assay), could be the alternative explanation for the higher response rates in all groups based on this assay. However, the MptD peptide response was statistically significant in the T1DM patients and it was not recognized in the controls (as low as in the 2% of people) confirming its high specificity. Moreover, when native M13 phage was used to investigate if the phage alone was recognized in T1DM, T2DM and controls, no statistically-significant results were found.

One of important observations that we recorded during this study was the absence of any significant association between MAP and the T2DM. T2DM had previously been linked to mycobacteria as early as in 1954 by Toba [28] and afterwards in 1964 [29] and recently by Broxmeyer *et al.* [30]. We here demonstrate that T2DM patients do not have significant levels of anti MAP antibodies in contrast to their T1DM counterparts. This seemingly hints at the possibility where the involvement of immune dysregulation (as seen in case of Crohn's disease and T1DM) in T2DM becomes irrelevant as a possible mechanism.

Given the satisfactory concordance of seropositivity and T1DM clinical phenotype, some concerns might originate from the observation of a slight positivity of MAP shown up in the control samples (as they were not 100% negative to the presence of anti MAP antibodies). We believe that fewer MAP positive control samples could represent subclinical cases of the past foci of MAP or they are genetically resistant to diabetes or Crohn's despite the fact that they have a defined load of MAP in their circulation.

As far as the application of the test is concerned, especially the MptD-based ELISA, we are confident that such tests hold significant potential for use in clinical diagnostics towards the species specific identification of MAP in T1DM and the Crohn's disease. Further, these tests might be used in equal success in the veterinary arena because there is no ‘gold standard’ herd level screening tool available for Johne's disease.

## Materials and Methods

### Patient and control plasma samples

A total of 136 participants comprising of 57 with T1DM and 79 healthy controls were tested for the presence of MAP specific antibodies. The healthy controls were randomly selected from the blood donors attending the University Hospital of Sassari. An equal number of type-2 diabetic (T2DM) patients (n = 57) were tested as compared to the same control individuals for the MptD peptide, fMptD and MAP antigen ELISAs. Written informed consents from patients and controls including other necessary clearances were obtained before blood samples were drawn. Institutional Review Board of the University of Sassari approved the study. In diabetic patients, clinical diagnoses for T1DM or T2DM were already confirmed including biochemical parameters such as glucose and insulin levels analyzed. Five millilitres of peripheral blood was drawn in heparinized vacutainer tubes from patients and controls and was centrifuged at low speed to separate plasma for use in ELISA. The same was aliquoted and stored frozen at −20°C for short-term storage (<6 months) and −80°C for long term storage (>6 months).

### Isolation of MAP after plasma inoculation into Mycobacterial selective media

Ten fresh samples from T1DM patients (from nos. 20 to 29), 10 samples from T2DM patients (from no. 20 to 29) and 10 control samples (from nos. 20 to 29) were further analysed for the presence of acid-fast bacilli after Ziehl-Neelsen staining of PMBC smears prepared from plasma. The samples were then inoculated into the tubes within a MGIT 960 non-radioactive culture system (Becton Dickinson) where Mycobactin J and Egg yolk emulsion (20 µl) had previously been added to the 7H10 medium (Becton Dickinson). The media were then incubated into the MGIT instrument until growth was detected or at 37°C in a 5% CO_2_ atmosphere for up to 6 months.

### MAP IS900 amplification

Mycobacterial DNA extraction and PCR to detect IS900 was performed as previously published [6], [18].

### ELISA with fMptD, MptD peptide and other MAP antigen (s) - HbHA, Gsd and MAP whole cell lysate

M13 phage fMptD, previously isolated [15] from the PhD-12 phage display library and was found to be specific for the MAP envelope protein MptD was obtained from the laboratory of Gerald F. Gerlach (University of Vet. Medicine, Hanover, Germany). MptD peptide (GKNHHHQHHRPQ) was synthesized by Brain Research Centre, UBC, 2211 Wesbrook Mall, Vancouver, BC, Canada V6T 2B5.

Recombinant proteins HbHA and Gsd and the whole cell lysate preparations were produced in our laboratories exactly as described previously [5], [12]. fMptD and MptD-peptide based ELISA were performed in 96-well microtitre plates (Maxisorp; Nunc-Immunoplate, Roskilde, Denmark). Each well was coated either with 10^8^ PFU of fMptD in carbonate bicarbonate buffer (Sigma-Aldrich) or, when MptD peptide was used, 50 µl of MptD peptide at the concentration of 5 µg/µl was spotted in each well and the microtitre plates were incubated at 4°C overnight. Next day, excess (of untithered) phage was washed out and each well was filled with 200 µl of blocking buffer (5% non-fat dried milk, Sigma-Aldrich) and the microtitre plates were incubated for 1 hour at room temperature (RT). After washing with 200 µl of PBS-T (PBS-0.05% Tween 20) for two times, 100 µl of diluted test plasma sample (1∶100 in PBS-T) was added to each well and incubated for 2 hours at room temperature. Plates were then washed five times with PBS-T and incubated for 1 hour with 100 µl of anti Human IgG-alkaline phosphatase antibody (Sigma–Aldrich) diluted to 1∶1000 in PBS-T. Five rounds of washings were performed and 200 µl of *p*-nitrophenylphosphate (Sigma–Aldrich) was added to each well and used as substrate for alkaline phosphatase (AP) enzyme. As the yellow color developed, plates were read at 405nm by using the VERSA Tunable Max microplate reader (Molecular Devices, USA). Phage Immuno Assay (fIA) and MptD-peptide immunoassay were also performed by using blood plasma as substrate to coat the microtitre plates (sensitivity obtained was lower than using the phage as a substrate, data not shown). However, the protocol using the MptD-peptide or the phage as a substrate as described above was found to be easy and efficient and was therefore, adopted as such and recommended for all the assays.

ELISA was also performed using the M13 phage as the only substrate against plasma of T1DM, T2DM patients and controls and no cross reaction was observed. ELISA with MAP antigens, HbHA, Gsd and whole cell lysates was performed as described previously [5]. Optical density values were compared with those obtained with MptD-peptide and fMptD assays above.

### Statistical analysis

All the ELISA, MptD-peptide and fIA experiments were performed in replicates and the results were expressed as means±the standard error (SE). All the values were compared using Chi-Square test of significance with Yate's corrections. All the values were also compared by a two-tailed Student's *t* test and considered significant if the *P* values were <0.05. *P* values were calculated using the online Graph Pad scientific calculator (http://www.graphpad.com_quickcalcs_ttest1.cfm).
